# German University Students’ Perspective on Remote Learning During the COVID-19 Pandemic: A Quantitative Survey Study With Implications for Future Educational Interventions

**DOI:** 10.3389/fpsyg.2022.734160

**Published:** 2022-02-24

**Authors:** Thomas Hoss, Amancay Ancina, Kai Kaspar

**Affiliations:** Department of Psychology, University of Cologne, Cologne, Germany

**Keywords:** COVID-19, higher education, remote learning, student perspective, study success, digital literacy, key study characteristics

## Abstract

The COVID-19 pandemic forced German universities to adjust their established operations quickly during the first nationwide lockdown in spring 2020. Lecturers and students were confronted with a sudden transition to remote teaching and learning. The present study examined students’ preparedness for and perspective on this new situation. In March and April 2020, we surveyed *n* = 584 students about the *status quo* of their perceived digital literacy and corresponding formal learning opportunities they had experienced in the past. Additionally, the students reported the direction of changes in key study characteristics they expected from this new situation. Moreover, they reported the extent to which they believe they will be able to master this new study situation successfully. Two categories of independent variables were considered: context-related variables and person-related variables. Our results show that students did not have many learning opportunities to promote their digital literacy, suggesting that they were not appropriately prepared for this new situation. Results for digital literacy vary by competence area. However, there is a positive correlation between past formal learning opportunities and corresponding digital competences. Master students reported more learning opportunities and higher digital literacy only in one competence area compared to bachelor students. Regarding the expected change of key study characteristics, some characteristics were expected to worsen and fewer to improve. A multiple regression analysis explained 54% of the estimated probability of successful remote learning. Students’ age, state anxiety, positive state affect, general self-efficacy, the availability of an own workplace, past learning opportunities in digital content creation, and the estimated preparedness of lecturers for remote teaching were significant explaining factors. Our results provide valuable insights into the perspective of students on studying during the COVID-19 pandemic and beyond. We discuss important factors that should be addressed by educational measures in the future.

## Introduction

The COVID-19 pandemic has strong impacts on people’s everyday life and society on a large scale (Nicola et al., 2020). During the first nationwide lockdown in Germany in spring 2020, educational institutions had to create remote teaching and learning environments in a very short time. Similarly, lecturers and students had to rapidly adjust their former concepts and approaches for teaching and learning (Shapiro et al., 2020). In general, the pandemic has significantly amplified the digital transformation of university teaching and learning. At the same time, university students’ express concerns about the impact of the COVID-19 pandemic on physiological, psychological, and educational issues (Branquinho et al., 2020). Therefore, the analysis of prerequisites, challenges, and expectations from a students’ perspective comes into focus. The present work pursued three objectives for addressing this student perspective:

First, it analyzes the *status quo* of students’ digital literacy and corresponding formal learning opportunities before the transition to remote learning. This analysis provides an assessment of whether students were adequately prepared for sudden remote learning and identifies areas of competence in which targeted support in formal university teaching would need to be strengthened.

Second, it explores the expectations of students regarding changes in key study characteristics associated with the transition to remote learning. This analysis is not only relevant as a historical classification, but also enables the identification of success and risk factors for good remote learning and can thus serve as a guideline for future measures to develop suitable learning opportunities.

Third, it examines a set of context- and person-related variables that may determine students’ estimated probability to master this new study situation of remote learning successfully. This analysis provides an estimate of the influence of various sources on the perceived likelihood of success and helps to prioritize target variables for educational interventions.

Importantly, at the time the survey was designed and conducted in March and April 2020, there was no COVID-19-related literature on the topic of digital learning. However, multiple studies providing important insights into students’ perspectives regarding digital learning during the pandemic have been published since then (e.g., Aristovnik et al., 2020; Krammer et al., 2020; Hamdan et al., 2021; Hawley et al., 2021). All these studies have in common that they have had to refer to research that predate the current pandemic. For this reason, the present study partially follows an exploratory approach. The corresponding results are highly relevant for the classification of the transformation processes in digital teaching and learning initiated at the beginning of the pandemic and serve as an important reference for the evaluation of the *status quo* as well as for the planning of future measures.

### Pre-pandemic Digital Literacy and Formal Learning Opportunities

To understand university students’ perspectives on learning in times of COVID-19, it is necessary to consider their prerequisites and prior experiences with remote learning. One essential prerequisite is digital literacy. Tang and Chaw (2016) identified digital literacy as an important factor for effective learning in digital learning environments. Digital literacy and ICT skills are important prerequisites for the successful participation of university students in learning processes (Shopova, 2014). Although students are familiar with technology and digital media, they might be more experienced in using technology for entertainment purposes than in the context of digital learning (Shopova, 2014; Prior et al., 2016). Past research also suggests that students sometimes overestimate their actual skills (Gross and Latham, 2012). Moreover, digital literacy is more than the ability to handle hardware and software properly. Just knowing the technology is not enough for successful learning (Coccoli et al., 2014). Digital literacy includes competences in reflective and critical thinking, management of information, and adequate online behavior (Tang and Chaw, 2016). The variety of different digital competences is described in the European Digital Competence Framework for Citizens (DigComp 2.1; Carretero et al., 2017). It consists of five competence areas: information and data literacy, communication and collaboration, digital content creation, safety, and problem-solving. These five areas are subdivided into 21 specific competences. Previous studies on digital literacy have already applied the DigComp framework to examine differences in digital competence areas and associated proficiency levels, for instance, between different generations (Khan and Vuopala, 2019), teachers and students (Kuzminska et al., 2018), or different European universities (López-Meneses et al., 2020). Khan and Vuopala (2019) found that competences in the area of problem solving were the least developed across all areas. In addition to individual competences, corresponding learning opportunities are an important prerequisite for digital learning, as they form the fundament for the acquisition of digital literacy. Indeed, prior studies showed that formal learning opportunities in study programs can have a positive impact on respective competences (König et al., 2018). However, digital media was often not an integral part of teaching and learning at universities before the pandemic (Persike and Friedrich, 2016). A recent pre-pandemic survey among students from a large German university indicated that learning opportunities to promote digital literacy are rather sparse or superficial, but their extent also varies across different competence areas (Jäger-Biela et al., 2020). Moreover, Jäger-Biela et al. (2020) found that master students report more learning opportunities in digital competence areas than bachelor students. However, for every of the examined competence areas more than half of the master students reported not having any learning opportunities in their studies. To better understand the initial situation at the beginning of the pandemic, we examined university students’ perceived digital literacy in terms of digital competences of the DigComp 2.1 and corresponding learning opportunities in formal university courses they had experienced (i.e. perceived) before the pandemic started. We asked:

RQ1: How do bachelor and master students evaluate the intensity of past formal learning opportunities and their level of competence in the respective areas, are there differences between competence areas, and are learning opportunities and competence assessments positively correlated?

### Expected Changes in Key Study Characteristics

Given the *status quo* of university students’ digital literacy and past formal learning opportunities, what were the expectations of students in spring 2020 regarding the upcoming semester, which was entirely based on remote teaching and learning? To get a more detailed picture, the following two questions need to be addressed: First, in which way will key study characteristics, such as the quality and quantity of learning materials or the support from other students, change? Second, on what factors does it depend whether students believe they can successfully master this new study situation?

From a students’ perspective, remote learning might be accompanied by a variety of advantages and disadvantages, compared to well-known face-to-face learning environments. For example, remote learning is connected to an increased flexibility in time management and the reception of course material (Daymont et al., 2011), and it may also foster self-regulated learning (e.g., Rüth et al., 2021). Also, remote learning may increase the quantity and quality of teaching and learning materials (Lin et al., 2017). In contrast, several disadvantages of remote learning can manifest such as a lack of interactions with peers and lecturers and less effective learning methods (Arkorful and Abaidoo, 2015). Also, shortcomings regarding technological infrastructure of universities could negatively impact study characteristics (cf. Gilch et al., 2019). In general, many study characteristics may change positively or negatively in the context of forced remote learning during a pandemic. Hence, we asked:

RQ2: What changes in key study characteristics are expected by university students?

### The Estimated Probability of Success

**FIGURE 1:**
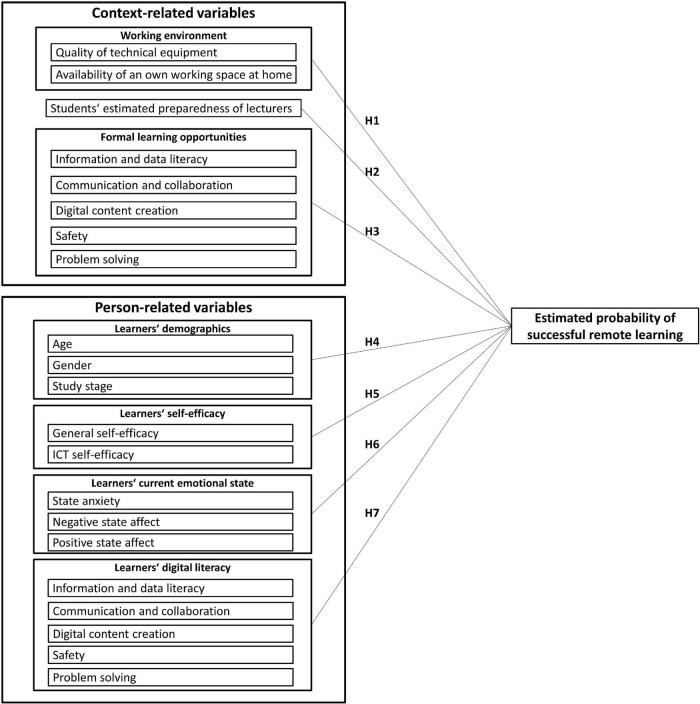
Context- and person-related variables and their hypothesized relation to students’ estimated probability of successful remote learning.

In addition to an analysis of expected changes in study characteristics due to a sudden transition to remote learning, the present study focused on factors that might explain interindividual differences in the belief that one can still learn successfully in this new situation (hereinafter referred to as “estimated probability of successful remote learning”). A person’s estimated probability of success is generally defined as the perceived probability of reaching a certain goal and it is dependent on the individual’s abilities (Zander and Heidig, 2020). The easier the goal is to achieve, the higher the person estimates his or her probability of success. In this study, we focused on basic context- and person-related variables. Figure 1 shows the corresponding research model with all its variables for which a relation to the estimated probability of success could be assumed on the basis of previous study results, as outlined in the following sections.

#### The Role of Context-Related Variables

##### Working Environment

When it comes to remote learning from home, students’ spatial- and technical infrastructure might influence their estimated probability of success. The immediate transition to remote learning makes the availability of an own adequate workspace and technical infrastructure necessary. Since students are forced to study at home, it is inevitable that their private space turns into a working space. In fact, “a suitable study desk located in a quiet area (preferably outside of the bedroom), free of distractions with plenty of natural light” (Brown et al., 2020, p. 24) was suggested as important factors for an appropriate learning environment. Alhabeeb and Rowley (2018) identified technical infrastructure, like internet access, communication tools, and their respective reliability, as an important success factor for digital learning. Indeed, students stated that an absence of technical infrastructure and an appropriate learning environment at home is problematic for studying during the COVID-19 pandemic (Kapasia et al., 2020). We hypothesized:

H1: The quality of technical equipment (H1a) and the availability of an own working space (H1b) are positively related to students’ estimated probability of successful remote learning.

##### Perceived Preparedness of Lecturers

Just like students, the abrupt transition to remote teaching and learning also posed challenges for university lecturers. The level of digital literacy and therefore the readiness for remote teaching differs among lecturers. One study reported that only every third teacher feels somewhat prepared to teach remotely (ElSaheli-Elhage, 2021). Additionally, the conception and execution of e-learning measures (Rüth and Kaspar, 2017), especially at the beginning, is time-consuming and depends on the experiences and skills of the lecturers (Tinker, 2001). McPherson and Nunes (2008) described staff issues, such as experience and availability of suitable lecturers, as a critical success factor for the delivery of e-learning. Paechter et al. (2010) found that students’ achievement goals and lecturer expertise are important predictors for knowledge, skill, and competence acquisition in e-learning courses. Variables like motivation, self-regulated and collaborative learning opportunities, as well as clarity of course structure also contributed significantly. In line with the above results, Joo et al. (2011) found that the characteristics of remote teaching influence students’ satisfaction. According to the authors, teachers should organize courses to enable active learning, conversation, and inclusion in the course. Perceived e-learning satisfaction can be predicted by interactive learning environments and seems to be related to perceived usefulness and self-regulation (Liaw and Huang, 2013). Thus, lecturers’ competences appear to have an important role for students’ study success and overall satisfaction with e-learning. Indeed, lecturers’ characteristics such as the ability to motivate students, their enthusiasm, and the ability to use e-learning systems effectively were considered as key factors for successful e-learning from the students’ perspective (Alhabeeb and Rowley, 2018). Hence, students’ perception of the lecturers’ preparedness in delivering adequate remote teaching might explain differences in their estimated probability of successful remote learning:

H2: The estimated preparedness of lecturers for remote teaching is positively related to students’ estimated probability of successful remote learning.

##### Formal Learning Opportunities

We also considered students’ past learning opportunities to promote digital literacy. Dealing with more demanding media applications requires more refined skills and learning opportunities (Pumptow and Brahm, 2020). In a nationwide study in Germany, learning with digital media was examined from a students’ perspective (Persike and Friedrich, 2016). Results showed that digital media are mainly used for private purposes. The use of digital media is concentrated in certain study programs, such as computer science and medicine. Similarly, Jäger-Biela et al. (2020) found that learning opportunities to promote digital literacy are rather sparse in university courses. This *status quo* seems to be critical, because students who have experienced formal learning opportunities more intensively might feel more prepared and perceive their probability of successful remote learning higher. We hence hypothesized:

H3: Experienced (i.e. perceived) learning opportunities to promote digital literacy are positively related to students’ estimated probability of successful remote learning.

#### The Role of Person-Related Variables

##### Learners’ Demographics

González-Gómez et al. (2012) found that female students score higher on average in e-learning courses, are more satisfied with e-learning, and assign more importance to teaching methods and planning than male students. In contrast, Ramírez-Correa et al. (2015) stated that the adaption of e-learning does not seem to depend on gender. Besides gender, Adams et al. (2018) found that younger students in higher education considered themselves as less independent learners. More specifically, Lai and Hong (2015) showed that they rely more on clear instructions and information before trying something new and seem to favor group work more than older students do. Nevertheless, there is no clear evidence that different age groups of students significantly vary in their use of digital technology and digital learning characteristics (Selwyn, 2008; Lai and Hong, 2015). Finally, cohort comparisons of bachelor and master students (pre-service teachers) indicated a better performance of the latter in all domains for didactic and pedagogical knowledge (König et al., 2018). Although comparable results on the development of digital literacy across different study stages are not yet available, digital literacy could also increase as students do progress through their study program. Given this mixed and incomplete research findings, we hypothesized in an undirected manner:

H4: Age (H4a) and gender (H4b) and study stage (H4c) are related to students’ estimated probability of successful remote learning.

##### Learners’ Self-Efficacy

The rising relevance of remote learning changed the accompanying demands students experience in higher education. These changing demands require students to adapt to the new situation. Self-efficacy is a personal belief about the self-evaluated competence of being able to handle such situations in a way to reach desired outcomes (Bandura, 1977) and it is a predictor of academic success (Zajacova et al., 2005). Students with higher perceived self-efficacy are more satisfied with e-learning university courses (Joo et al., 2013). Furthermore, self-efficacy has a significant impact on learning achievement, which in turn significantly affects learning persistence. However, in an exceptional pandemic situation, university students’ perception of academic self-efficacy might be reduced (Alemany-Arrebola et al., 2020). Besides a general dimension, self-efficacy should also be evaluated concerning the specific domain (Pajares, 1996; Klassen and Chiu, 2010). For the domain of remote learning, ICT self-efficacy showed a positive relation to achievements in the area of computer and information literacy (Rohatgi et al., 2016). A more recent study revealed positive correlations between students’ ICT self-efficacy and motivation, goal orientation, interest, and study success, but a negative correlation with anxiety (Pumptow and Brahm, 2020). This study also found that self-assessed e-learning skills, like application use and programming, are positively correlated with digital media self-efficacy. Hence, we hypothesized:

H5: General self-efficacy (H5a) and ICT self-efficacy (H5b) are positively related to students’ estimated probability of successful remote learning.

##### Learners’ Current Emotional State

According to Liaw and Huang (2013), perceived e-learning satisfaction can be predicted by perceived self-efficacy and perceived anxiety. However, the authors pointed out that the negative relation between perceived anxiety and perceived satisfaction is relatively small and anxiety may not be the most significant predictor. Nevertheless, results of a longitudinal study showed that difficulties at university, like financial or relationship problems, can increase students’ anxiety and depression levels (Andrews and Wilding, 2004). Because of the ongoing COVID-19 pandemic, perceived anxiety could have an even stronger impact. COVID-19 related research showed that there are major psychological health problems among university students during phases of lockdowns (Cao et al., 2020; Rodríguez-Hidalgo et al., 2020), including symptoms such as anxiety, stress, and depression. However, students also appear to be able to deal with anxiety during the pandemic (Baloran, 2020). Therefore, the current emotional state of students should be considered with respect to their estimated probability of successful remote learning. We hence hypothesized:

H6: There is a relation between state anxiety (H6a), negative state affect (H6b), and positive state affect (H6c) on the one hand, and students’ estimated probability of successful remote learning on the other.

##### Learners’ Digital Literacy

In the context of universities and the ongoing digitization in higher education, digital literacy is an important factor for successful learning: Students are more and more required to navigate within the digital landscape, that is, being proficient in various software programs and in handling digital tools sufficiently (Koc and Bakir, 2010), but also being able to critically reflect digital technology (e.g., Rüth and Kaspar, 2020). According to Jimoyiannis (2015), digital literacy not only includes elements of ICT literacy, but also “a variety of knowledge, attitudes, and complex skills which people need to function effectively in contemporary digital environments” to be able to acquire, critically use, and create further knowledge (Hagel, 2015; p. 4). Digital literacy incorporates computer, internet, information, visual, and media literacy (Jimoyiannis, 2015). Therefore, digital literacy is a prerequisite for skill acquisition and successful learning (Tang and Chaw, 2016), in and beyond higher education (Littlejohn et al., 2012; Techataweewan and Prasertsin, 2018). Given the increasing digital and technological requirements within higher education and the importance of digital literacy for academic success, we hypothesized:

H7: Digital literacy is positively related to students’ estimated probability of successful remote learning.

## Materials and Methods

### Participants

This study is the second part of a larger survey conducted in April and May 2020. Participants were recruited by a combination of convenience and snowball sampling methods. The final sample used for statistical analyses included 584 university students (496 female, 82 male, and 6 diverse). Conditions of participation were a minimum age of 18 years and enrollment at a German university. Students enrolled at distance-learning universities were excluded. Age of the students ranged from 18 to 66 years with a mean of 24.07 years (*SD* = 4.88). Most of the students (*n* = 403) were in a bachelor’s degree program, 181 were studying for a master’s degree. The sample contained students of different study programs: 404 students participated in one of several teacher education programs covering a wide range of scientific disciplines, 71 were studying psychology, and 38 were studying a media-oriented program. Participation was voluntary and anonymous. Incentives to participate were not provided.

### Measures

The survey started with demographic questions including age, gender, study program, number of semesters studied, and name of the university enrolled in. The questionnaire was administered in German language. Participants were informed in advance that they could terminate their participation at any time without giving reasons and that their data would then not be included in the study. Hence, the final data set contains only participants who provided a complete dataset.

#### Digital Literacy and Past Formal Learning Opportunities

The assessment of students’ digital literacy and corresponding learning opportunities was based on the DigComp 2.1 (Carretero et al., 2017). This framework contains five competence areas and 21 competences, each described in a short statement. Based on these statements, we created 21 one-sentence-items to circumscribe each competence. A detailed overview of the used items is displayed in Supplementary Table A. Students reported the intensity with which they had learned these competences by means of past learning opportunities within their study program (1 = not at all, 5 = very intensively). Additionally, students were asked to rate their level of competence (1 = very low, 5 = very high). The first competence area deals with “information and data literacy” and contains three competences (e.g., “Analyze, compare, and critically evaluate data, information, and digital content and their sources.”). We calculated a composite score for this competence area by averaging across the items for learning opportunities (α = 0.75) as well as for perceived competence (α = 0.75). The second competence area deals with “communication and collaboration” and contains six competences (e.g., “Collaborate with others using digital technologies and co-create resources and knowledge.”). Cronbach’s α was 0.87 for learning opportunities and 0.84 for perceived competence. The third competence area focuses on “digital content creation” and contains four competences (e.g., “Create and edit digital content and be able to express oneself through digital means”). Cronbach’s α was 0.74 for learning opportunities as well as for perceived competence. The fourth competence area is about “safety” and contains four competences (e.g., “Protect technical devices and digital content and understand risks and threats in digital environments”). Cronbach’s α was 0.83 for learning opportunities and 0.80 for perceived competence. The fifth competence area focuses on “problem solving” and contains four competences (e.g., “Identify and solve technical issues while operating devices and using digital environments”). Cronbach’s α was 0.87 for learning opportunities and 0.82 for perceived competence.

#### Expected Changes in Key Study Characteristics

Students were asked to estimate how several study characteristics would change in light of the transition to remote teaching and learning. Specifically, we asked them to estimate the potential change in 12 study characteristics compared to their study experience before the pandemic (see “Results” Section). A response scale ranging from −2 (= deteriorating) over 0 (= no change) to +2 (= improving) was used.

#### Estimated Probability of Successful Remote Learning

To assess students’ belief that they can successfully study in the new remote setting, we used the scale “probability of success” of the Questionnaire on Current Motivation (QCM; Rheinberg et al., 2001). This scale contains four items (α = 0.80) assessing learners’ probability of success (e.g., “I believe to be up to the challenge of this task” and “I probably won’t be able to successfully complete the task”). We slightly adapted the introduction so that the items refer to the new study situation of remote learning. The ratings were given on a five-point scale ranging from 1 (= does not apply) to 5 (= applies).

#### Quality of Technical Equipment and Availability of an Own Working Space at Home

To determine whether the students had the necessary spatial and technical resources to successfully take part in remote teaching and learning, the existence of four characteristics were rated: sufficiently fast and stable internet connection, required software, required hardware, and own permanent learning space. The answer options “No, I do not own” and “I do not know exactly” were coded as zero, the answer option “Yes, I do own” was coded as one. It should be noted that in order to achieve the highest possible test power in the later multiple regression model, we refrained from coding response “I do not know exactly” as missing data, as this would not affect the results regarding this variable. The first three items were aggregated to a sum score indicating the quality of students’ technical equipment for remote learning, the last item served as dummy-coded variable indicating the availability of an own working place at home. The complete items and English translations can be found in the Supplementary Table B.

#### Students’ Estimated Preparedness of Lecturers for Remote Teaching

The abrupt transition to remote teaching caused by the pandemic also poses unexpected challenges for lecturers. We asked the students to give an overall evaluation of the lecturers they have met so far in their study program. Specifically, the students assessed their lecturers’ skills, motivation, and consideration of student needs with respect to remote teaching: “What percentage of your previous lecturers do you think, based on your experience, are capable of realizing a good, entirely digital learning environment?”, “What percentage of your previous lecturers do you think, based on your experience, are motivated to realize a good, entirely digital learning environment?”, and “What percentage of your previous lecturers do you think, based on your experience, will consider students’ interests and needs when realizing an entirely digital learning environment?”. The original items in German language can be found in the Supplementary Table B. Ratings were given on a 11-point scale ranging from 0 (= 0%) to 10 (= 100%). We computed a composite score to assess the estimated preparedness of lecturers for remote teaching by averaging across the three items (α = 0.85).

#### General Self-Efficacy and ICT Self-Efficacy

We used a German short form of the Self-efficacy Scale to assess general self-efficacy (AKSU; Beierlein et al., 2012). The scale (α = 0.88) comprises three items (e.g., “In difficult situations I can rely on my skills”) and uses a five-point Likert scale (1 = totally disagree, 5 = totally agree).

ICT self-efficacy refers to beliefs held while using information and communications technology for various learning purposes. A scale developed by Siddiq et al. (2017) was used to measure ICT self-efficacy. This scale (α = 0.83) comprises three items (e.g., “I am sure I know how to collaborate with other students by use of digital technology”), which were rated on a five-point Likert scale (1 = totally disagree, 5 = totally agree).

#### State Anxiety

To measure state anxiety, we used a German short scale (α = 0.87) of the State-Trait Anxiety Inventory (STAI-SKD, Englert et al., 2011). Students rated the items (e.g., “I am tense,” and “I am concerned”) according to their current emotional state with respect to the forthcoming remote learning semesters. Hence, the instruction was slightly adapted asking: “How do you feel with regard to the forthcoming online semester?”. We used a five-point scale (1 = not at all, 5 = a lot).

#### Positive and Negative State Affect

State affect was measured by means of the German version of the Positive and Negative Affect Schedule (PANAS; Krohne et al., 1996). The students used a five-point scale (1 = not at all, 5 = extremely) to rate 20 adjectives describing different feelings and sensations (e.g., “active,” “interested,” “distressed,” and “scared”). The students rated how they felt this way during the past few days. Cronbach’s α for positive affect was 0.86, and it was 0.84 for negative affect.

## Results

### Digital Literacy and Past Formal Learning Opportunities (RQ1)

We analyzed the *status quo* regarding digital learning in terms of self-rated digital competences and past learning opportunities experienced in the formal study program. As shown in Table 1, the intensity of past learning opportunities was rated as low overall. An ANOVA for repeated measures (Greenhouse–Geisser applied) was computed to compare the intensity ratings across competence areas (within-participant comparisons based on the identical measurement scale). We found a significant effect, *F*(3.06, 1782.24) = 378.67, *p* < 0.001, $\eta_{\text{p}}^{2}$ = 0.39. According to pairwise comparisons (Bonferroni-adjusted), the intensity of perceived learning opportunities in the area “information and data literacy” was higher than the intensity of all other learning opportunities. As shown in Table 1, most comparisons were significant, all *p*s < 0.001, except for “communication and collaboration” versus “digital content production,” *p* = 0.053, and “safety” versus “problem solving,” *p* = 0.854.

**TABLE 1:** Descriptive statistics and bivariate correlations between perceived learning opportunities and self-rated digital literacy.

DigComp 2.1 competence area	Perceived learning opportunities		Perceived competence		Bivariate correlation	
	*M*	*SD*	*M*	*SD*	*r*	*p*
Information and data literacy	2.61*^a^*	0.87	3.41*^a^*	0.74	0.39	<0.001
Communication and collaboration	1.91*^b^*	0.77	3.42*^a^*	0.77	0.31	<0.001
Digital content creation	1.85*^b^*	0.69	2.53*^b^*	0.74	0.52	<0.001
Safety	1.58*^c^*	0.73	2.93*^c^*	0.84	0.37	<0.001
Problem solving	1.63*^c^*	0.75	2.68*^d^*	0.85	0.46	<0.001

*Perceived competences were measured on a scale ranging from 1 (very low) to 5 (very high). Perceived learning opportunities were measured on a scale ranging from 1 (not at all) to 5 (very intensive). Mean values with different superscripts (a–d) indicate statistically significant differences between competence areas (Bonferroni-adjusted significance level, all ps ≤ 0.001).*

In contrast to learning opportunities, perceived competences were rated as moderate, that is, around the scale’s midpoint. An ANOVA for repeated measures (Greenhouse–Geisser applied) compared the self-ratings across competence areas. Again, we found a significant effect, *F*(3.66, 2133.43) = 353.99, *p* < 0.001, $\eta_{\text{p}}^{2}$ = 0.38. As shown in Table 1, significant differences between all but one competence areas existed, all *p*s < 0.001 (Bonferroni-adjusted), except the contrast “information and data literacy” versus “communication and collaboration,” *p* > 0.999.

Moreover, we found significant positive correlations between competence ratings and respective past learning opportunities for each competence area (Table 1). Thus, the more intensive the formal learning opportunities were, the more students felt competent in that particular area.

To better assess the generalizability of the results, we next examined possible differences at the level of selected subgroups. On the one hand, we focused on study stage by comparing bachelor and master students. As shown in Table 2, master students rated their learning opportunities and digital competences significantly higher with respect to competence area “information and data literacy.” However, in all other competence areas, there was no difference in either the competence assessment or the prior learning opportunities. Importantly, these results still persisted when a homogenous group of bachelor students studying a specialized media-oriented program (called Intermedia) were excluded. Indeed, we exploratively compared this group (*n* = 36) with bachelor students (*n* = 33) and master students (*n* = 38) who study psychology. We selected these three groups as they were of similar size and can be considered as relatively homogenous regarding study content and course of study, in contrast to the strong heterogeneity in the rest of the sample. We found no group difference regarding perceived learning opportunities and self-rated competence in the area “information and data literacy.” For all other competence areas, Intermedia students stated having more learning opportunities than bachelor and master psychology students. Group differences were less pronounced with respect to competences: Intermedia students (vs. psychology students) attributed higher competences to themselves in competence area “problem solving.” For competence area “safety,” Intermedia students considered their competence higher than master psychology students but not significantly higher than bachelor psychology students. Strikingly, no differences were found in learning opportunities and competences between bachelor and master psychology students. To sum up, in the case of perceived learning opportunities in particular, there were significant differences between the subgroups in favor of those students in whose study program media use and production occupies a central place, albeit at an overall low absolute level. Competences are rated as moderate on average, but only few group differences existed. Detailed results can be found in the Supplementary Table C.

**TABLE 2:** Comparison between master and bachelor students regarding perceived learning opportunities and self-rated digital literacy.

DigComp 2.1 competence area	Perceived learning opportunities				*t*-test			Perceived competence				*t*-test		
				
	Master (*n* = 181)		Bachelor (*n* = 403)		*t*	*p*	*d*	Master (*n* = 181)		Bachelor (*n* = 403)		*t*(582)	*p*	*d*
	*M*	*SD*	*M*	*SD*				*M*	*SD*	*M*	*SD*			
Information and data literacy	2.75	0.88	2.55	0.86	2.56	0.011	0.23	3.62	0.66	3.32	0.76	4.54	<0.001	0.41
Communication and collaboration	1.84	0.70	1.95	0.80	–1.56	0.120	–0.13	3.44	0.75	3.42	0.78	0.33	0.738	0.03
Digital content creation	1.86	0.71	1.84	0.68	0.30	0.762	0.03	2.60	0.69	2.51	0.75	1.36	0.173	0.12
Safety	1.58	0.69	1.58	0.75	–0.05	0.958	–0.00	2.92	0.88	2.94	0.83	–0.26	0.798	–0.02
Problem solving	1.58	0.70	1.64	0.77	–0.97	0.331	–0.08	2.73	0.86	2.66	0.84	0.90	0.368	0.08

*Perceived competences were measured on a scale ranging from 1 (very low) to 5 (very high). Perceived learning opportunities were measured on a scale ranging from 1 (not at all) to 5 (very intensive).*

### Expected Changes in Key Study Characteristics (RQ2)

In the next step, we analyzed students’ estimation of how twelve different study characteristics would change considering the transition to remote teaching and learning. The rating scale ranged from −2 (= deteriorating) to +2 (= improving). Table 3 shows descriptive statistics, results of one-sample *t*-tests comparing the observed mean value with the scale’s midpoint (0 = no change), and the frequency distribution of ratings. Results indicate that students did not expect deterioration in all areas of study. In numbers, four of the twelve characteristics were expected to significantly improve, namely the quantity and quality of learning materials provided online by lecturers, the possibility of a self-defined learn and time schedule, and temporal possibilities for undisturbed individual learning. In contrast, seven study characteristics were expected to worsen, namely students’ access to relevant literature, the mutual supportiveness among students, the availability of lecturers, the quality of communication between students and lecturers, the general learning environment, students’ personal identification with their studies, and their collaboration with other students in the context of lectures and seminars. The largest effect size (*d* = −1.33) was observed for the latter study characteristic. No change was expected regarding the spatial possibilities for undisturbed individual learning, due to the frequency of answers being distributed evenly across the possible answers. In general, the frequency distributions differed remarkably across study characteristics, but for each characteristic, there were both students who expected improvements and students who expected deterioration due to remote teaching and learning.

**TABLE 3:** Descriptive statistics, one sample *t*-tests, and frequency distributions for expected changes in key study characteristics.

Variable	*M*	*SD*	One-sample *t*-test			Frequency distribution in %				
			*t*(583)	*p*	*d*	−2	−1	0	+1	+2
Quantity of learning materials provided by lecturers	0.71	1.11	15.37	<0.001	0.64	4.79	9.93	22.26	35.62	27.40
Quality of learning materials provided by lecturers	0.42	0.95	10.69	<0.001	0.44	2.74	10.62	42.47	30.31	13.87
Students’ access to relevant literature	–0.50	1.27	–9.60	<0.001	–0.40	28.77	24.14	23.29	16.27	7.53
Collaboration with other students in the context of lectures and seminars	–1.18	0.89	–32.10	<0.001	–1.33	44.01	36.30	14.73	4.11	0.86
Mutual supportiveness among students	–0.20	1.11	–4.45	<0.001	–0.18	13.87	25.00	35.62	18.66	6.85
Availability of lecturers	–0.09	1.02	–2.07	0.039	–0.09	8.39	26.54	35.27	25.00	4.79
Quality of communication between students and lecturers	–0.51	1.03	–11.94	<0.001	–0.49	16.44	38.70	26.71	15.41	2.74
Possibility of a self-defined learn and time schedule	0.84	1.11	18.23	<0.001	0.75	3.42	11.13	17.29	34.25	33.90
Spatial possibilities for undisturbed, individual learning	–0.09	1.37	–1.57	0.117	–0.06	18.49	24.14	24.14	14.21	19.01
Temporal possibilities for undisturbed, individual learning	0.65	1.18	13.35	<0.001	0.55	6.85	9.93	21.75	34.42	27.05
General learning environment	–0.40	1.18	–8.25	<0.001	–0.34	17.98	36.13	22.43	15.24	8.22
Students’ personal identification with their studies	–0.43	1.02	–10.19	<0.001	–0.42	15.92	29.79	40.58	8.73	4.97

*One-sample t-tests were computed against the scale’s midpoint of 0. The sum of the percentage values might differ from 100% due to rounding.*

### Explaining Students’ Estimated Probability of Successful Remote Learning (H1 – H7)

The final analysis addressed students’ belief about their ability to successfully master the new remote learning situation created by the abrupt transition to remote learning due to the pandemic, see Figure 1. Six participants reported their gender as “diverse” and were excluded because this was an insufficient subsample for the following blockwise regression analysis: Initially, context-related independent variables were considered as a first block in the regression model (Model 1, Table 4). Subsequently, person-related variables were added (Model 2). Students’ estimated probability of successful remote learning served as the dependent variable.

**TABLE 4:** Bivariate correlations and results of blockwise multiple regression analysis for students’ estimated probability of successful remote learning as dependent variable.

	Bivariate correlation		Model 1			Model 2		
	*r*	*p*	*B*	ß	*p*	*B*	ß	*p*
Constant			1.86			2.29		
Quality of technical equipment	0.25	<0.001	0.17	0.15	0.001	0.04	0.04	0.243
Availability of own working space	0.25	<0.001	0.43	0.20	<0.001	0.28	0.13	<0.001
Preparedness of lecturers for remote learning	0.39	<0.001	0.13	0.33	<0.001	0.08	0.19	<0.001
Information and data literacy OTL	0.20	<0.001	0.10	0.11	0.009	0.06	0.06	0.094
Communication and collaboration OTL	0.15	<0.001	0.02	0.02	0.769	0.03	0.03	0.470
Digital content creation OTL	0.11	0.009	–0.10	–0.09	0.092	–0.19	–0.17	<0.001
Safety OTL	0.11	0.011	0.06	0.05	0.309	0.03	0.03	0.501
Problem solving OTL	0.12	0.004	0.03	0.03	0.655	0.07	0.07	0.176
Age	–0.03	0.459				–0.01	–0.07	0.014
Gender (0 = male, 1 = female)	0.03	0.532				0.13	0.06	0.076
Study stage (0 = bachelor, 1 = master)	0.07	0.086				0.08	0.05	0.165
General self-efficacy	0.50	<0.001				0.24	0.25	<0.001
ICT self-efficacy	0.49	<0.001				0.05	0.06	0.228
State anxiety	–0.56	<0.001				–0.25	–0.34	<0.001
Negative state affect	–0.43	<0.001				–0.01	–0.01	0.775
Positive state affect	0.37	<0.001				0.13	0.12	<0.001
Information and data literacy competence	0.33	<0.001				0.00	0.00	0.986
Communication and collaboration competence	0.39	<0.001				0.01	0.01	0.803
Digital content creation competence	0.30	<0.001				0.10	0.09	0.072
Safety competence	0.25	<0.001				–0.03	–0.03	0.530
Problem solving competence	0.30	<0.001				–0.03	–0.03	0.497
*R^2^/R^2^_*adj*_*			0.24/0.23			0.54/0.52		

*The analysis was based on n = 578 since participants how reported their gender as “diverse” (n = 6) were not included; Model 1 includes all context-related variables; Model 2 includes all context-related variables and all person-related variables. p-values are based on bootstrapping with 5,000 iterations; OTL = opportunities to learn.*

The intercorrelations-matrix of all independent variables is presented in the Supplementary Table D. The correlations were rather low, with few exceptions. Ratings of different learning opportunities showed the most pronounced intercorrelations (*r*_*max*_ = 0.70). Besides, general self-efficacy and ICT self-efficacy (*r* = 0.65) as well as state anxiety and negative affect (*r* = 0.61) showed rather high correlations, indicating construct validity.

All statistical assumptions of the multiple regressions were checked (cf. Poole and O’Farrell, 1971), and most of them were met. Normality assumption was given by visual means, however, the Shapiro–Wilk test was significant. Additionally, because specific forms of heteroscedasticity can be hardly detected *via* visual inspection and statistical tests, we used bootstrapping (5,000 iterations) to ensure unbiased significance tests (Hayes and Cai, 2007).

Table 4 shows the results of the blockwise regression analysis. In the first model, limited to context-related variables, the estimated preparedness of lecturers, the availability of an own working space, the quality of technical equipment, and prior learning opportunities regarding information and data literacy showed a positive relation to the estimated probability of success. Learning opportunities in other competence areas did not show a significant relation. Overall, Model 1 explained 24% of the interindividual variance in the estimated probability of successful remote learning, *F*(8, 569) = 22.60, *p* < 0.001. Model 2 added person-related variables and increased the explanatory power of the model to 54% explained variance, *F*(21, 556) = 31.00, *p* < 0.001. In this complete model, the availability of an own working space (H1b) and the estimated preparedness of lecturers for remote teaching (H2) still showed significant positive relations, but quality of technical equipment (H1a) and learning opportunities for information and data literacy did not anymore. However, learning opportunities regarding digital content creation revealed a significant negative relation to the estimated probability of success: The more intensive the corresponding learning opportunities were, the lower was the estimated probability of successful remote learning. Remaining learning opportunities did not show a significant relation to probability of successful remote learning (H3). Moreover, age did show a negative relation to the probability of success (H4a), whereas gender (H4b) and study stage (H4c) did not. Interestingly, general self-efficacy (H5a), but not ICT self-efficacy (H5b), was positively related to the estimated probability of successful remote learning. Students’ state anxiety, but not negative state affect (H6b), showed a significant negative relation to the estimated probability of successful remote learning. In contrast, positive state affect (H6c) was positively related to the probability of success. Importantly, and contradicting our expectations, none of the five domain-specific competence ratings showed a relation to the estimated probability of successful remote learning (H7). However, on the level of bivariate correlations, all competence ratings showed a significant positive relation to the estimated probability of successful remote learning. Hence, when competences were taken into account simultaneously and other variables were added, the multiple regression yielded a different picture. In general, all independent variables showed the expected significant bivariate correlation to the dependent variable (H1 – H7), except age, gender, and study stage (H4). However, these relationships only partially held in the multiple regression. The three most important independent variables were – according to standardized regression coefficients in Model 2 and in descending order – state anxiety, general self-efficacy, and the estimated preparedness of lecturers for remote teaching.

## Discussion

The early stages of the COVID-19 pandemic and the sudden transition to remote learning evoked a variety of challenges for university students in Germany. The present study was a timely response to this situation, and it pursued three objectives: We analyzed the *status quo* of students’ digital literacy and corresponding (past) learning opportunities at the beginning of the transition to remote learning. Additionally, we examined the expectations of students regarding changes in study characteristics. Finally, we examined a set of context- and person-related variables that may determine students’ estimated probability to master this new study situation of remote learning successfully. We observed several important findings, which we will discuss in the next sections.

### Digital Literacy and Past Formal Learning Opportunities

The results show that digital literacy in terms of self-rated competences and their respective past formal learning opportunities were positively correlated across competence areas. We also found that the extent of these competences differed among areas, with digital content production having the lowest self-rating. This result is noteworthy because the DigComp 2.1 (Carretero et al., 2017) treats these areas as five equal parts of one dimension. However, it should be taken into account that self-reports are sometimes biased and do not always reflect true competence levels (Aesaert et al., 2017). Similarly, the students’ reported that formal learning opportunities to promote digital literacy had been rather sparse before the pandemic began, but their intensity also significantly varied across competence areas. Nevertheless, there appears to be a systematic lack of relevant learning opportunities in university courses. Indeed, earlier works have already highlighted that digital media were rarely part of teaching and learning in German universities before the pandemic (Persike and Friedrich, 2016) and that the *status quo* of digitalization was considered to be improvable (Gilch et al., 2019). Undoubtedly, forced remote teaching and learning during the pandemic has reinforced the need for a comprehensive digital transformation process.

Given the discrepancy between the self-assessment of moderate competences and the low intensity of corresponding learning opportunities in university courses, we may speculate that students acquire significant parts of digital competences outside the formal university context. However, it is important to note that students’ general media use does not necessarily correlate with performance in digital learning (Persike and Friedrich, 2016). Nonetheless, these results may be a solid basis for universities and lecturers to implement measures and content to support underrepresented areas of learning opportunities to promote digital literacy. Also, these findings could serve as a basis for future research related to the COVID-19 pandemic and remote learning in general. A key question is whether the quantity and quality of formal learning opportunities has changed over the course of the pandemic and whether students rate their competences higher after going through a long period of enforced distance learning. It is important to observe whether formal learning opportunities to promote digital literacy and related skills have increased and whether any changes are only short-term or long-term.

Bachelor and master students only differed significantly in their perceived learning opportunities and digital competence in the area of “information and data literacy.” The effect sizes even for the significant differences are rather small. This result contradicts prior studies (Jäger-Biela et al., 2020) where master students reported more learning opportunities across all competence areas. However, it is noteworthy that Jäger-Biela et al. (2020) used a different competence framework and their sample only consisted of students enrolled in different teacher education programs. The role of individual study programs for learning opportunities is supported by our explorative analysis: Students of a media-oriented study program reported significantly more learning opportunities compared to bachelor and even master students in psychology. Interestingly, the differences in self-assessed competences were less pronounced, with students in the media-oriented program reporting higher competences in two of five competency areas, namely “safety” and “problem solving.” Nevertheless, and similar to the overall sample, media students’ mean scores for prior formal learning opportunities and perceived competences were at most in the middle range of the scale. This shows that there is basically still a lot of room for improvement for all students and that corresponding offers for competence trainings should be pushed across all study programs.

### Expected Changes in Key Study Characteristics

The advantages and disadvantages of remote learning have been largely covered in research before (Arkorful and Abaidoo, 2015). Our results provide a novel perspective on the impact of the sudden transition to remote learning on study characteristics. While students expected seven out of twelve key study characteristics to worsen on average, they still believed that four study characteristics would improve. This distribution is in line with the perception of an overall more negative than positive impact of the pandemic (Petzold et al., 2020). Students felt an increase in anxiety and a fear of social isolation in the early stages of the pandemic (Benke et al., 2020; Cao et al., 2020). Especially the fear of social isolation is visible in our results. Collaboration with other students in lectures and seminars as well as the quality of communication between students and lecturers were the two aspects where students expected the most deterioration, indicating that communication and social interaction are perceived as being less likely in the digital sphere (cf. Masoumi and Lindström, 2012). These results underline that many students need social support and interaction in their studies, which should be addressed by adequate measures. The access to relevant literature was also expected to worsen, indicating an overdependence of German universities on presence services and operations, and thus indicating a lack of digitalization (Gilch et al., 2019). Moreover, from the perspective of personal and professional development, it is a warning signal that the students expected that their personal identification with their studies would deteriorate significantly.

However, our results also showed that the overall quality and quantity of learning materials provided online by lecturers was expected to improve. Furthermore, the possibility of a self-defined learning and time schedule as well as temporal possibilities for undisturbed individual learning were two further characteristic that were expected to improve. In this context, the central question is whether students actually possess the skills necessary to take advantage of increased flexibility and self-regulated learning. Consequently, universities should create specific measures that promote the necessary skills for self-regulated learning while maintaining students’ personal identification with their studies. Noteworthy, the spatial possibilities for undisturbed, individual learning were not expected to change (on average). However, the expectations of students spread evenly across the range of possible answers, indicating substantial inter-individual variance in the quality of learning places at home.

### Explaining Students’ Estimated Probability of Successful Remote Learning

Given that individual optimism may play such a central role when dealing with remote learning under pandemic circumstances, we deepened the analyses in this respect. We focused on factors that might explain why students are more or less optimistic regarding the belief that one can nevertheless successfully master the new remote learning situation. For this purpose, we focused on context-related variables and person-related variables. Our model explained a substantial amount of 54% inter-individual variance. As expected, most of these factors were found to play a significant role. Importantly, we refer to the results of the regression model in the following section, instead of bivariate correlations. In order to assess the significance of the individual factors, their joint rather than separate contribution should be considered.

#### The Role of Context-Related Variables

##### Working Environment

Interestingly, the quality of students’ own technical equipment in terms of internet connection, required software, and required hardware for remote learning only showed a significant relation to students’ estimated probability of successful remote learning when limiting the analysis to context-related variables. When person-related variables were added, this relation changed to being non-significant. In developing countries, the lack of internet access and adequate technology poses a problem for remote learning during the COVID-19 pandemic (Adnan and Anwar, 2020). In Germany, where the present study took place, a vast majority of students are sufficiently equipped technologically, so this aspect seems to play a subordinate role for successful remote learning. In contrast, the availability of an own working space was positively related to the students’ estimated probability of successful remote learning in both models. The relevance of an optimal environment at home that enables and stimulates remote learning has not really been the subject of research so far. In the situation of forced remote learning during the COVID-19 pandemic, the lack of a suitable workspace is especially problematic as it could further contribute to a spiral where already socially disadvantaged students might be disadvantaged even more. This has implications for policy makers and universities to help ensuring that certain students do not fall too far behind. Future research should focus more on the characteristics of home learning spaces.

##### Preparedness of Lecturers

One of the most important factors was students’ perceived preparedness of lecturers for remote teaching. It seems to be a success factor from a student perspective if lecturers are capable and motivated to create remote teaching, while taking the interests of the students into account. Students who indicated that a greater percentage of their lecturers are able to meet these criteria reported a higher probability of successful remote learning. This finding is consistent with lecturer characteristics being a critical success factor for digital learning (Alhabeeb and Rowley, 2018). However, the lecturers are also put to the test by the sudden switch to remote teaching, as there was no time for the required training and to gain the necessary experiences and skills. A study by Krammer et al. (2020) investigated the perspective of university students on online courses and remote learning during the COVID-19 pandemic. Their results indicate that active involvement by lecturers, clearly structured tasks, and lecturers’ feedback are positive factors for remote teaching and learning. What can be learned from the pandemic and the present results is that lecturers should be intensively trained in the adequate use of digital resources. Future research should consider the lecturers’ perspective and characteristics accordingly.

##### Formal Learning Opportunities

Lastly, past learning opportunities to promote digital literacy were not a positive contributor to students’ estimated probability of successful remote learning. The competence area of digital content creation showed a significant relation, but in negative direction. This result is surprising and a consequence of the simultaneous consideration of several factors and their (low to moderate) intercorrelations. Indeed, on the level of bivariate correlations, all learning opportunities showed positive relations to the probability of successful remote learning. However, it is not implausible that the more intensive the learning opportunities were in terms of digital content creation, the less likely success in remote learning was rated. Presumably, students who have experience with digital content creation are aware of the procedural and time-related problems related to the development and implementation of remote teaching and learning materials. They might be more aware of the challenges associated with the sudden transition elicited by the pandemic, and consequently think that their probability of successful remote learning is lower. In general, the topic of learning opportunities will become increasingly important during the pandemic and beyond. Our study was conducted at the beginning of the pandemic, and digital learning opportunities may have grown in the last four semesters since then. In addition, this could help identifying which learning opportunities are the most important and need to be implemented in a sustainable manner in different study programs. However, our results also indicate that besides formal learning opportunities, other variables need to be considered.

#### The Role of Person-Related Variables

##### The Role of Learners’ Demographics

Previous research reported mixed results regarding the impact of gender and age on digital learning (e.g., González-Gómez et al., 2012; Lai and Hong, 2015; Ramírez-Correa et al., 2015; Adams et al., 2018). In the context of the COVID-19 pandemic, gender did not show an effect regarding the attitude to remote learning (Dikaya et al., 2021). In the present study, gender and study stage (bachelor versus master program) did not play a significant role, but age did. The older students were the lower was the estimated probability of success in remote learning. One possible explanation is that older students have developed more established learning routines over their previous lifespan that were disrupted by the COVID-19 pandemic, resulting in less flexibility in adapting to the new situation. The fact that these routines are less applicable in the new situation could have had a negative impact on their assessment of success. In general, it might be easier to create new strategies for remote learning than adapting prior established strategies from face-to-face settings. Indeed, Millar et al. (2021) stated that new learning strategies emerged among first semester university students. Other recent research on remote learning during the COVID-19 pandemic suggests that age and study stage, as well as gender, should not be considered alone and are only a few of a variety of potentially relevant sociodemographic variables (Vladova et al., 2021). More important variables might be the income of students, as it might influence the availability of specific technological infrastructure at home, and the individual family background, as this can take on a supporting or a burdening function in times of a pandemic. Indeed, studies showed that the pandemic has led to job losses or reduced income (Aucejo et al., 2020) as well as to new obligations and challenges in family life (Ayuso et al., 2020).

##### The Role of Learners’ Self-Efficacy

Our results support the finding that self-efficacy is positively related to academic success (Zajacova et al., 2005). General self-efficacy was positively related to the probability of successful remote learning. However, we did not find a significant role of the more specific ICT self-efficacy. A current study found significant relations between internet self-efficacy and students’ satisfaction with online education in times of the COVID-19 pandemic (Hamdan et al., 2021). One reason might be that the probability of successful remote learning in an online semester covers much more than dealing with technology-related study characteristics and was therefore related to the more global concept of general self-efficacy in the present study. Interestingly, Heo et al. (2021) recently examined the structural relationship between different domains of self-efficacy and online learning engagement. They found that self-efficacy in technology use itself did not increase learning engagement. Self-efficacy in an online learning environment, however, had an influential role. Therefore, it seems fruitful that future research should use more than one domain of self-efficacy and examine the relations between these domains. Furthermore, time management and self-regulation (Hamdan et al., 2021; Heo et al., 2021) seem to be relevant factors for remote learning and should be considered when examining remote learning in the future.

##### The Role of Learners’ Current Emotional State

Besides self-efficacy as a general belief and demographic variables, we focused on students’ current emotional states in this pandemic. Strikingly, the level of state anxiety was the most influential independent variable regarding the estimated probability of successful remote learning. The more anxiety the students reported, the lower their scores were on the outcome variable. Importantly, negative state affect did not show a significant relation, suggesting that the more specific emotion of anxiety is a more suitable indicator here. Indeed, the whole situation elicited by the pandemic and the first nationwide lockdown have led to anxiety, distress, and uncertainty among German adults in general (Benke et al., 2020; Petzold et al., 2020). When we asked students about their state of anxiety regarding the upcoming remote learning semester, they had to consider an unpredictable long time in the future. As a consequence, students were dealing with uncertainty regarding their course of studies and the development of the pandemic in general. In contrast, state affect referred only to the past few days. Nonetheless, positive state affect was positively related to the students’ estimated probability of successful remote learning. Overall, the results clearly show that the emotional sphere is an important factor, but one that is not typically addressed through targeted interventions within formal university teaching. A rethinking of this point, at least in times of an exceptional situation for society as a whole, should be considered.

##### The Role of Learners’ Digital Literacy

Like learning opportunities, all areas of digital literacy showed a significant positive bivariate correlation with the probability of successful remote learning. However, combined with context-related variables and other person-related variables, competences did not play a significant role for the estimated probability of successful remote learning. While under normal circumstances digital literacy might be a prerequisite for successful learning (Tang and Chaw, 2016), the situation during the COVID-19 pandemic might be perceived as different. Although a majority of students seemed to be confident about their digital competences and skills during COVID-19 lockdowns (Tejedor et al., 2020), in a new and unknown situation characterized by anxiety and distress (Benke et al., 2020; Petzold et al., 2020) the level of anxiety and affect might overweight the existence of digital literacy. Moreover, it is also possible that the ability to efficiently and appropriately adapt existing competences to new situational conditions is more important than simply expanding competences. In any case, it is crucial that educational interventions to improve digital literacy in the university context are complemented by interventions to manage anxiety and stress and to improve adaptability in relation to new learning situations.

### Limitations

This study provides a detailed analysis of the immediate perspective of German university students on remote learning during the COVID-19 pandemic. The data reflect the *status quo* during the initial phase of the pandemic but provides numerous implications for future action. Nevertheless, there are also limiting factors for the results and implications that should be considered:

First of all, the sample consists of mostly female university students enrolled in several teacher education programs with different scientific disciplines, psychology, or a media-oriented program. The vast majority studied at the same large German university. The sample was obtained through an unsystematic combination of convenience sampling and snowball sampling. Hence, this sample is not representative for all German university students. Relatedly, because the study started at the beginning of a new semester, it is completely unclear to what extent the participants had previously experienced the same learning opportunities and to what extent corresponding measurements might be characterized by some dependency. The immediacy of the national lockdown and transition to remote teaching and learning made it impossible to specify and trace appropriate subgroups. At least, a selected subgroup analysis indicated a limited variability of results and hence some generalizability across study programs. In principle, however, the mean differences between universities or study programs could be smaller than between some parallel courses within a study program if their lecturers implement distance learning in completely different ways or dramatically differ in their own digital literacy. Hence, future research should specifically focus on the variability of learning opportunities and competence distributions across different institutional levels.

Also, one limitation is the fact that all measurements were self-reports. While these are necessary for some constructs (e.g., state anxiety and self-efficacy), more objective measurements for learning opportunities and real competence levels would be desirable. However, instruments that allow objective measurement of digital literacy are very sparse and do not cover the wide range of competences outlined in the DigComp 2.1 Framework. Moreover, an objective analysis of formal learning opportunities is very difficult (cf. Jäger-Biela et al., 2020), because the mention of certain contents in official course descriptions does not provide any information about whether the intended curriculum was actually implemented and realized in this way.

Another possible limitation might be that the present study design was cross-sectional and only covered the perspective of students on remote learning. Therefore, no causal relations can be drawn and the perspective of teachers on remote teaching remains unclear. More measuring points or a post-course evaluation of student’s actual success were not part of this study. At the beginning of the study, it was unknown how long the lockdown and associated measures would last, which is why a longitudinal approach was (unfortunately) not taken into account. Lastly, another limitation is the explorative approach of our study. Our regression model was solely based on previous empirical evidence and a simple distinction between context- and person-related variables, as existing and more elaborated models usually did not fit well to the specific situational demands of the pandemic situation. It must also be kept in mind that at the early time of the study, no pandemic-related educational research had been published, and many of the papers cited here were occurring concurrently. Since then, more and more literature addressing the students’ perspective on remote learning was published (e.g., Krammer et al., 2020; Tejedor et al., 2020; Hamdan et al., 2021. Hawley et al., 2021). Thus, there are generally few references to relevant (i.e., pandemic-related) prior work in the current literature, but all of this work, taken together, provides a valuable resource for planning future research and practical measures. In this regard, this study also contributes an important complementary piece to the literature.

### Conclusion

Overall, this study shows that students lacked formal learning opportunities to promote digital literacy at the early stages of the pandemic. It also revealed that formal learning opportunities are positively related to students’ digital literacy, which was rated as moderate. Differences between bachelor and master student were only found in one competence area. Although learning opportunities seem to vary significantly across selected study programs, there is basically still a lot of room for improvement, both in terms of learning opportunities and related competences. The sudden transition to remote learning led to specific expectations regarding changes of key study characteristics, in negative and positive directions. We found that both context- and person-related variables are relevant in explaining students’ estimated probability of success in remote learning. The proposed model showed remarkable explanatory power and provides a solid foundation for future research and further elaborated models. General self-efficacy, an own working space, current anxiety, positive state affect, students’ age, and the estimated preparedness of lecturers for remote learning were identified as relevant variables explaining the perceived probability of success. Importantly, perceived digital literacy and four out of five corresponding learning opportunities did not show a significant relation to this key outcome variable when considered simultaneously with all other contextual and personal variables. In summary, these results show possible starting points for measures to improve digital learning and teaching in the long term.

## Data Availability Statement

The original contributions presented in the study are included in the article/Supplementary Material, further inquiries can be directed to the corresponding author.

## Ethics Statement

Ethical review and approval was not required for the study on human participants in accordance with the local legislation and institutional requirements. In Germany, as stated by the German Research Association (DFG, https://www.dfg.de/foerderung/faq/geistes_sozialwissenschaften/index.html), the present survey study did not require the approval of an ethics committee, because the research did not pose any threats or risks to the respondents, it was not associated with high physical or emotional stress, and the respondents were informed about the objectives of the survey in advance. At the beginning of the study, participants were informed that the data of this study will be used for research purposes only and that all data are collected anonymously. Thus, no identifying information was collected. Participants who prematurely abandoned the survey were not included in the analyses and all of their data were deleted from the dataset. The patients/participants provided their written informed consent to participate in this study. Informed consent to participate in this study was provided by clicking a corresponding box, and participation was voluntary in all cases.

## Author Contributions

TH, AA, and KK designed the study, performed the analyses, interpreted the results, and wrote the manuscript. TH and AA collected the data. KK organized and supervised data collection and acquired funding. All authors contributed to the article and approved the submitted version.

## Conflict of Interest

The authors declare that the research was conducted in the absence of any commercial or financial relationships that could be construed as a potential conflict of interest.

## Publisher’s Note

All claims expressed in this article are solely those of the authors and do not necessarily represent those of their affiliated organizations, or those of the publisher, the editors and the reviewers. Any product that may be evaluated in this article, or claim that may be made by its manufacturer, is not guaranteed or endorsed by the publisher.
