# Supplementary material for: German University Students’ Perspective on Remote Learning During the COVID-19 Pandemic: A Quantitative Survey Study With Implications for Future Educational Interventions
Source: Front Psychol. 2022 Feb 24;13:734160. doi: 10.3389/fpsyg.2022.734160 (PMC8907854; doi:10.3389/fpsyg.2022.734160)
Supplement: Supplementary file 1 [file Data_Sheet_1.PDF]

Supplementary Material

Supplementary Table A

Overview of original German items for digital learning opportunities and competences based on the DigComp 2.1 and respective English translations.

Variable	German item	English item
Competences and learning opportunities (based on DigComp 2.1)	**Umgang mit Informationen und Daten**<br>1. Recherchieren, suchen und filtern von Daten, Informationen und digitalen Inhalten.<br>2. Analysieren, vergleichen und kritisches Bewerten von Daten, Informationen und digitalen Inhalten sowie deren Quellen.<br>3. Organisieren, speichern und abrufen von Daten, Informationen und Inhalten in digitalen Umgebungen.	**Information and data literacy**<br>1. Browse, search and filter data, information, and digital content.<br>2. Analyze, compare, and critically evaluate data, information, and digital content and their sources.<br>3. Organize, store, and retrieve data, information, and content in digital environments.
	**Kommunikation und Zusammenarbeit**<br>1. Interagieren mit anderen Personen mittels digitaler Technologien und angemessene Formen digitaler Kommunikation für einen gegebenen Kontext auswählen.<br>2. Teilen von Daten, Informationen und digitalen Inhalten mit anderen Personen über dafür angemessene digitale Technologien.<br>3. Teilnehmen am gesellschaftlichen Leben durch die Nutzung von öffentlichen und privaten digitalen Services.<br>4. Zusammenarbeiten mit anderen Personen mittels digitaler Technologien und gemeinsam Ressourcen und Wissen erzeugen.<br>5. Beachten von Verhaltensnormen und Regeln während der Nutzung digitaler Technologien und der Interaktion mit anderen Personen in digitalen Umgebungen (Netiquette).<br>6. Erstellen und managen einer oder mehrerer digitaler Identitäten und schützen der eigenen digitalen Reputation.	**Communication and collaboration**<br>1. Interact with others using digital technologies and select appropriate forms of digital communication for a given context.<br>2. Share data, information, and digital content with others through appropriate digital technologies.<br>3. Participate in society through using public and private digital services.<br>4. Collaborate with others using digital technologies and co-create resources and knowledge.<br>5. Be aware of behavioral norms and know-how while using digital technologies and interact with others in digital environments (netiquette).<br>6. Create and manage one or multiple digital identities and protect one's own digital reputation.
	**Erzeugen digitaler Inhalte**<br>1. Erstellen und bearbeiten von digitalen Inhalten und sich über digitale Wege ausdrücken können.<br>2. Modifizieren, weiterentwickeln, verbessern und integrieren von Informationen und digitalen Inhalten.<br>3. Wissen, welcher Kopierschutz und welche Lizenzen für Daten, Informationen und digitale Inhalte gelten.<br>4. Planen und entwickeln einer Sequenz von verständlichen Instruktionen für einen Computer, damit dieser ein Problem löst oder eine spezielle Aufgabe ausführt (Programmierung).	**Digital content creation**<br>1. Create and edit digital content and be able to express oneself through digital means.<br>2. Modify, refine, improve, and integrate information and digital content.<br>3. Understand what copy protection and licenses apply to data, information, and digital content.<br>4. Plan and develop a sequence of understandable instructions for a computing system to solve a problem.

Supplementary Table A (continued)

German item	English item
**Sicherheit**<br><br>1. Schützen von technischen Geräten und digitalen Inhalten sowie Risiken und Bedrohungen in digitalen Umgebungen verstehen.<br>2. Schützen von personenbezogenen Daten und der Privatsphäre.<br>3. Vermeiden von Gesundheitsrisiken und Bedrohungen für das geistige und körperliche Wohlbefinden während der Nutzung von digitalen Technologien.<br>4. Wissen um den Einfluss von digitalen Technologien und ihrer Nutzung auf die Umwelt.	**Safety**<br><br>1. Protect technical devices and digital content and understand risks and threats in digital environments.<br>2. Protect personal data and privacy.<br>3. Avoid health risks and threats to psychological and physical well-being while using digital technologies.<br>4. Be aware of the environmental impact of digital technologies and their use.
**Problemlösen**<br><br>1. Identifizieren und lösen technischer Probleme während der Bedienung von Geräten und der Nutzung von digitalen Umgebungen.<br>2. Ermitteln von Bedürfnissen und identifizieren, bewerten, auswählen und nutzen von angemessenen digitalen Werkzeugen zu deren Befriedigung.<br>3. Anwenden von digitalen Werkzeugen und Technologien, um neues Wissen und innovative Prozesse und Produkte zu erzeugen.<br>4. Verstehen, an welcher Stelle die eigene digitalisierungsbezogene (Medien-) Kompetenz verbessert und auf den neuesten Stand gebracht werden muss.	**Problem solving**<br><br>1. Identify and solve technical issues while operating devices and using digital environments.<br>2. Assess needs and identify, evaluate, select, and use appropriate digital tools to meet them.<br>3. Use digital tools and technologies to create new knowledge and innovate processes and products.<br>4. Understand where one's own digital competence needs to be improved or updated.

Note. The items used in the questionnaire were in German language. The respective English translations are only provided for international readability. Perceived competences were measured on a scale ranging from 1 (*very low*) to 5 (*very high*). Experienced learning opportunities were measured on a scale ranging from 1 (*not at all*) to 5 (*very intensive*).

Supplementary Table B

Overview of items estimating quality of technical equipment, own working space, and preparedness of lecturers for remote teaching.

Variable	German item	English item
Quality of technical equipment	1. Ausreichend schnelle und stabile Internetverbindung.<br>2. Notwendige Software (mindestens Programme für Textverarbeitung, Präsentationen und digitale Kommunikation).<br>3. Notwendige Hardware (mindestens eigenen Laptop/PC mit Mikrofon und Kamera).	1. Sufficiently fast and stable internet connection.<br>2. Necessary software (at least programs for word processing, presentations, and digital communication).<br>3. Necessary hardware (at least own laptop/PC with microphone and camera).
Availability of own working space	1. Eigenen permanenten Lernplatz (mindestens eigenen Schreibtisch).	1. Own permanent learning space (at least own desk).
Students' estimated preparedness of lecturers for remote teaching	1. Wie viel Prozent Ihrer bisherigen Dozierenden trauen Sie auf Basis Ihrer gemachten Erfahrungen die Fähigkeit zu, einen guten, vollständig digitalen Lehrbetrieb realisieren zu können?<br>2. Wie viel Prozent Ihrer bisherigen Dozierenden schätzen Sie auf Basis Ihrer gemachten Erfahrungen als motiviert ein, einen guten, vollständig digitalen Lehrbetrieb realisieren zu wollen?<br>3. Wie viel Prozent Ihrer bisherigen Dozierenden werden Ihrer Einschätzung nach studentische Interessen und Bedürfnisse bei der Realisierung eines vollständig digitalen Lehrbetriebs ausreichend berücksichtigen?	1. What percentage of your previous lecturers do you think, based on your experience, are capable of realizing a good, entirely digital learning environment?<br>2. What percentage of your previous lecturers do you think, based on your experience, are motivated to realize a good, entirely digital learning environment?<br>3. What percentage of your previous lecturers do you think, based on your experience, will consider students' interests and needs when realizing an entirely digital learning environment?

Note. The items used in the questionnaire were in German language. The respective English translations are only provided for international readabili

Supplementary Table C

Descriptive and inferential statistics of perceived learning opportunities and perceived competences for selected study programs.

**DigComp 2.1 competence area**	**Perceived learning opportunities**			**ANOVA**			**Perceived competence**			**ANOVA**		
	Media-oriented Bachelor (n = 36)	Psychology Bachelor (n = 33)	Psychology Master (n = 38)	F	p	η^2	Media-oriented Bachelor (n = 36)	Psychology Bachelor (n = 33)	Psychology Master (n = 38)	F	p	η^2
	M (SD)	M (SD)	M (SD)				M (SD)	M (SD)	M (SD)			
Information and data literacy	2.79^a (0.84)	2.62^a (0.90)	2.85^a (0.84)	0.70	.500	.01	3.53^a (0.67)	3.33^a (0.74)	3.63^a (0.65)	1.70	.188	.03
Communication and collaboration	2.33^a (0.79)	1.87^b (0.71)	1.78^b (0.54)	6.77	.002	.12	3.73^a (0.55)	3.40^a (0.66)	3.57^a (0.58)	2.53	.085	.05
Digital content creation	2.29^a (0.76)	1.86^b (0.71)	1.82^b (0.61)	5.11	.008	.09	2.83^a (0.83)	2.72^a (0.79)	2.63^a (0.54)	0.71	.495	.01
Safety	2.23^a (0.95)	1.68^b (0.82)	1.73^b (0.67)	4.86	.010	.09	3.27^a (0.67)	2.89^{ab} (0.87)	2.74^b (0.72)	4.75	.011	.08
Problem solving	2.19^a (0.85)	1.70^b (0.81)	1.55^b (0.61)	7.29	.001	.12	3.22^a (0.72)	2.61^b (0.79)	2.51^b (0.68)	9.93	< .001	.16

Note. Perceived competences were measured on a scale ranging from 1 (*very low*) to 5 (*very high*). Experienced learning opportunities were measured on a scale ranging from 1 (*not at all*) to 5 (*very intensive*). Mean values with different superscripts (a-b) indicate statistically significant differences between the study programs for each competence area of learning opportunities and competences separately (Bonferroni-adjusted significance level, all $ps \leq .032$).

Supplementary Table D

Bivariate correlations (Pearsons’ *r* and two-tailed *p*) among all variables included in the regression model.

**Variable**	1.	2.	3.	4.	5.	6.	7.	8.	9.	10.	11.
1. Quality of technical equipment											
2. Availability of own working space	0.20***										
3. Preparedness of lecturers for remote learning	0.15***	0.09*									
4. Information and data literacy OTL	0.11**	0.02	0.22***								
5. Communication and collaboration OTL	0.08	–0.01	0.26***	0.48***							
6. Digital content creation OTL	0.08	–0.04	0.24***	0.47***	0.70***						
7. Safety OTL	0.05	–0.10*	0.15***	0.37***	0.61***	0.61***					
8. Problem solving OTL	0.04	–0.02	0.19***	0.38***	0.66***	0.69***	0.70***				
9. Age	0.02	–0.06	–0.03	0.06	0.01	0.01	0.00	–0.04			
10. Gender	–0.03	0.02	0.09*	–0.05	0.01	–0.00	–0.02	–0.04	–0.02		
11. Study stage	0.02	–0.03	–0.19***	0.10*	–0.06	0.01	–0.00	–0.04	0.25***	0.00	
12. General self-efficacy	0.22***	0.14***	0.18***	0.11**	0.05	0.07	0.03	0.02	0.12**	–0.08*	0.18***
13. ICT self-efficacy	0.33***	0.14***	0.27***	0.23***	0.21***	0.23***	0.18***	0.18***	0.01	–0.08	0.09*
14. State Anxiety	–0.19***	–0.12**	–0.26***	–0.11**	–0.09*	–0.10*	–0.05	–0.02	0.00	0.05	–0.06
15. Negative state affect	–0.19***	–0.14***	–0.24***	–0.10*	–0.06	–0.04	0.01	–0.01	0.02	0.08*	–0.09*
16. Positive state affect	0.08*	0.06	0.19***	0.17***	0.18***	0.12**	0.15***	0.19***	0.08	–0.02	0.07
17. Information and data literacy competence	0.18***	0.14**	0.13**	0.38***	0.19***	0.23***	0.19***	0.18***	0.04	–0.06	0.19***
18. Communication and collaboration competence	0.23***	0.09*	0.25***	0.21***	0.31***	0.30***	0.20***	0.24***	–0.01	–0.05	0.02
19. Digital content creation competence	0.18***	0.02	0.19***	0.24***	0.32***	0.52***	0.36***	0.39***	0.06	–0.10*	0.06
20. Safety competence	0.18***	0.04	0.14**	0.18***	0.20***	0.28***	0.37***	0.26***	–0.07	–0.10*	–0.01
21. Problem solving competence	0.18***	0.07	0.18***	0.20***	0.30***	0.37***	0.31***	0.46***	–0.03	–0.18***	0.04
22. Estimated probability of successful remote learning	0.25***	0.25***	0.39***	0.20***	0.15***	0.11**	0.11*	0.12**	–0.03	0.03	0.07

Supplementary Table D (continued)

**Variable**	12.	13.	14.	15.	16.	17.	18.	19.	20.	21.
13. ICT self-efficacy	0.65***									
14. State Anxiety	–0.30***	–0.40***								
15. Negative state affect	–0.32***	–0.34***	0.61***							
16. Positive state affect	0.31***	0.25***	–0.26***	–0.27***						
17. Information and data literacy competence	0.44***	0.51***	–0.22***	–0.23***	0.20***					
18. Communication and collaboration competence	0.45***	0.58***	–0.33***	–0.31***	0.24***	0.53***				
19. Digital content creation competence	0.37***	0.50***	–0.24***	–0.17***	0.20***	0.50***	0.61***			
20. Safety competence	0.34***	0.40***	–0.22***	–0.17***	0.18***	0.39***	0.55***	0.61***		
21. Problem solving competence	0.39***	0.51***	–0.24***	–0.20***	0.22***	0.47***	0.57***	0.70***	0.63***	
22. Estimated probability of successful remote learning	0.50***	0.49***	–0.56***	–0.43***	0.37***	0.33***	0.39***	0.30***	0.25***	0.30***

Note. OTL = opportunities to learn (learning opportunities), Gender was dummy-coded (0 = male, 1 = female), study stage was dummy-coded (0 = bachelor, 1 = master), availability of own working space was dummy-coded (0 = no, 1 = yes), All results are based on n = 578. ***p < .001; **p < .01; *p < .05
